# Vasoconstrictor responsiveness through alterations in relaxation time and metabolic rate during rhythmic handgrip contractions

**DOI:** 10.14814/phy2.13933

**Published:** 2018-12-03

**Authors:** Jacob T. Caldwell, Shelbi L. Sutterfield, Hunter K. Post, Garrett M. Lovoy, Heather R. Banister, Shane M. Hammer, Carl J. Ade

**Affiliations:** ^1^ Department of Kinesiology Kansas State University Manhattan Kansas

**Keywords:** Blood flow, duty cycle, functional sympatholysis, vascular control

## Abstract

Increasing the relaxation phase of the contraction–relaxation cycle will increase active skeletal muscle blood flow (${\dot{Q}}_{m}$). However, it remains unknown if this increase in ${\dot{Q}}_{m}$ alters the vasoconstriction responses in active skeletal muscle. This investigation determined if decreasing mechanical impedance would impact vasoconstriction of the active skeletal muscle. Eight healthy men performed rhythmic handgrip exercise under three different conditions; “low” duty cycle at 20% maximal voluntary contraction (MVC), “low” duty cycle at 15% MVC, and “high” duty cycle at 20% MVC. Relaxation time between low and high duty cycles were 2.4 sec versus 1.5 sec, respectively. During steady‐state exercise lower body negative pressure (LBNP) was used to evoke vasoconstriction. Finger photoplethysmography and Doppler ultrasound derived diameters and velocities were used to measure blood pressure, forearm blood flow (FBF: mL min^−1^) and forearm vascular conductance (FVC: mL min^−1^ mmHg) throughout testing. The low duty cycle increased FBF and FVC versus the high duty cycle under steady‐state conditions at 20% MVC (*P *< 0.01). The high duty cycle had the greatest attenuation in %ΔFVC (−1.9 ± 3.8%). The low duty cycle at 20% (−13.3 ± 1.4%) and 15% MVC (−13.1 ± 2.5%) had significantly greater vasoconstriction than the high duty cycle (both: *P *< 0.01) but were not different from one another (*P* = 0.99). When matched for work rate and metabolic rate ($\dot{V}O_{2}$), the high duty cycle had greater functional sympatholysis than the low duty cycle. However, despite a lower $\dot{V}O_{2}$, there was no difference in functional sympatholysis between the low duty cycle conditions. This may suggest that increases in ${\dot{Q}}_{m}$ play a role in functional sympatholysis when mechanical compression is minimized.

## Introduction

During steady‐state rhythmic exercise, the majority of muscle blood flow (${\dot{Q}}_{m}$) is delivered during the relaxation phase of the contraction–relaxation cycle (i.e., duty cycle) (Broxterman et al. 2014; Bentley et al. 2017). Thus, increasing duty cycle (i.e., increasing mechanical impedance) may decrease ${\dot{Q}}_{m}$ due to a shorter relaxation phase (Hoelting et al. 2001). As such, increasing duty cycle has been shown to reduce the capacity to sustain a set power output during handgrip exercise (Broxterman et al. 2014; Bentley et al. 2017). This is thought to be attributed to increases in mechanical impedance, which will result in increased muscle fatigue and compromised exercise capacity (Broxterman et al. 2014; Bentley et al. 2017). Conversely, lowering the duty cycle for a given contraction frequency will result in less mechanical impedance and increased oxygen delivery (Bentley et al. 2017). Thus, the lower duty cycle will increase ${\dot{Q}}_{m}$, consequently leading to higher sustainable aerobic power outputs (Broxterman et al. 2014).

While it is known that the increased relaxation period facilitates greater O_2_ delivery to the active skeletal muscle, it remains unclear if decreasing the mechanical impedance (increased relaxation), can impact sympathetically mediated vasoconstriction (i.e., functional sympatholysis) (Remensnyder et al. 1962; Thomas et al. 1994; Joyner and Thomas 2003). One may speculate that decreasing mechanical impedance will impact functional sympatholysis in active skeletal muscle via changes in the internal milieu of the contracting muscle (i.e., decreased metabolite production and accumulation) (Thomas et al. 1994; Tschakovsky et al. 2002; Kruse et al. 2017). Recent work by Kruse et al. 2017 demonstrated that slower contraction–relaxation frequencies (1:2 sec) were less likely to maintain ${\dot{Q}}_{m}$ when sympathetic outflow was increased. However, their use of a slower contraction frequency versus duty cycle not only increased relaxation time, but decreased metabolic rate as well, which would also impact vasoconstriction if less glycolytic fibers were recruited (Thomas et al. 1994). An example of altering relaxation time with matched metabolic rate is best shown by Bentley et al. (2017), in that they used an inflatable cuff to lengthen mechanical impedance of the duty cycle, reducing relaxation time and ${\dot{Q}}_{m}$. They showed a greater rebound in ${\dot{Q}}_{m}$ during the higher duty cycle (i.e., shorter relaxation) versus the control condition to offset the increase in mechanical impedance. The rebound in ${\dot{Q}}_{m}$ during the reduced relaxation phase indicated a compensatory vasodilation to support greater ${\dot{Q}}_{m}$ and suggests that metabolite buildup may play a key role in metabolic vasodilation (Bentley et al. 2017). As such, altering duty cycle without changes to contraction frequency may provide additional insight on mechanical impedance and functional sympatholysis (Hamann et al. 2005).

To the best of our knowledge, there are no current reports that have examined if increasing the relaxation phase during handgrip exercise alters functional sympatholysis during lower body negative pressure (LBNP) evoked vasoconstriction. Therefore, the purpose of the current investigation was to test the hypotheses that a low duty cycle (i.e., decreased mechanical impedance) would elicit a larger vasoconstriction during increased sympathetic outflow compared to a high duty cycle (i.e., increased mechanical impedance) at a matched work rate. Furthermore, given that the majority of evidence has demonstrated that metabolic rate dictates functional sympatholysis, we also hypothesized that a lower work rate with increased relaxation time would elicit a larger vasoconstrictor response than the matched duty cycle. From this information we can begin to investigate the relationship between in duty cycle and vasoconstriction responses.

## Methods

### Participants

Eight healthy, recreationally active, men (age 25 ± 2 years [mean ± SE]; height 177 ± 1 cm; mass 84 ± 5 kg) volunteered to participate in the current investigation. All participants reported to the laboratory after a minimum 3‐h fast and were asked to avoid heavy exercise for 24 h or caffeine and alcohol prior to data collection. Based on a physical activity questionnaire, all participants completed <5 total hours of recreational activity per week. All experimental procedures and methods were approved by the Institutional Review Board of Kansas State University and conformed to the standards set forth by the *Declaration of Helsinki*. Prior to data collection all subjects signed an informed consent and filled out a health history screening form for overt diseases (e.g., cardiovascular, metabolic, renal). All testing was completed in a temperature‐controlled laboratory (20−22°C) at the same time of day.

### Experimental measurements

Beat‐by‐beat mean arterial pressure (MAP) was measured via finger photoplethysmography (Finometer Pro, FMS, the Netherlands) and calibrated to brachial artery blood pressure according to manufacturer specifications. Measurements of brachial artery diameter and blood velocity were simultaneously measured with an ultrasound system (LOGIQ S8, GE medical systems, Milwaukee, WI) equipped with a multi‐frequency linear array transducer operating at 10 MHz and placed ~10 cm proximal to the antecubital fossa with care taken to avoid the bifurcation of the artery. All measurements had a Doppler sample volume set at the full width of the vessel with the insonation angle <60° and were captured during both rest and exercise. Brachial artery images were stored offline and diameters were analyzed using a commercially available edge‐detection and wall‐tracking software package (Vascular research tools 6, [Medical Imaging Applications, Coraville, IA]) as described previously (Caldwell et al. 2016).

### Near‐infrared spectroscopy

Microvascular heme concentrations (i.e., hemoglobin + myoglobin) were measured with a frequency domain multidistance near‐infrared spectroscopy (NIRS) probe (OxiplexTS, ISS, Champaign, IL) that was placed longitudinally over the flexor digitorum superficialis of the exercising arm and had a black cloth placed over the site to limit light, described in detail previously (Craig et al. 2017). Briefly, the NIRS probe consists of a detector fiber bundle, four light‐emitting diodes, and operates at wavelengths of 690 and 830 nm (source‐detector distance 2.5–4.0 cm). The NIRS device allows for absolute (*μ*mol/L) quantification of total‐[heme] and deoxygenated heme concentration (deoxy‐[heme]). Importantly, because the NIRS device cannot dissociate myoglobin from hemoglobin, the term [heme] is used herein. The NIRS probe was calibrated prior to each test using a phantom block supplied by the manufacturer. Identification of the muscle belly was identified by a single experienced investigator palpating during muscle contraction and remained in position throughout testing. The NIRS data were collected throughout the protocol at 50 Hz, stored for post hoc analysis, and time aligned with the blood pressure and blood flow data.

### Lower body negative pressure

Subjects were placed into a custom‐built LBNP chamber in a supine position. Once subjects were in the chamber, an initial test to familiarize and confirm a proper seal of the chamber was performed. The level of LBNP (~30 mmHg) used in the current investigation has been confirmed previously to primarily unload the cardiopulmonary baroreceptors without altering arterial pressure and provides reproducible increases in muscle sympathetic nerve activity in the forearm (Hansen et al. 1996; Fadel et al. 2004; Vongpatanasin et al. 2011). LBNP was used at rest and during steady‐state exercise for 2‐min to allow resting and exercising vasoconstriction comparisons.

### Experimental protocol

**Figure 1:**
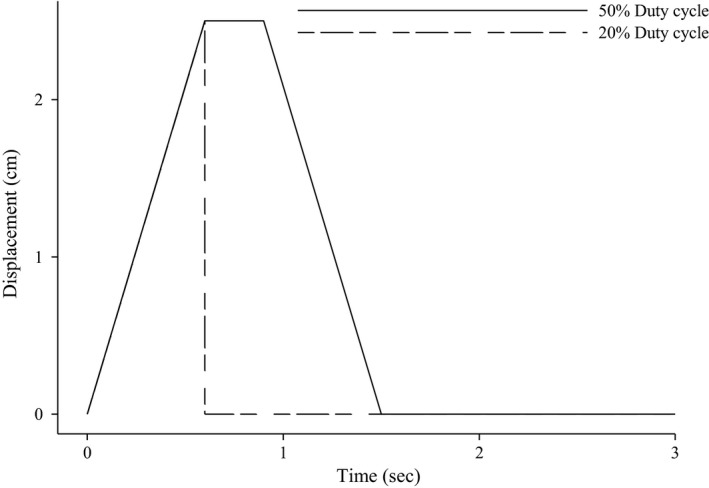
Duty cycle paradigm. The low duty cycle (20%) matches the high duty cycle (50%) concentric contraction time (0.6 sec) and excludes the isometric transition phase (0.3s), and the eccentric relaxation phase (0.6 sec).

Testing was performed while subjects were supine in a custom‐built LBNP chamber based on previously reported specifications (Esch et al. 2007). All exercise was performed by dynamically contracting a custom‐built two‐pillar handgrip dynamometer with a maximal displacement of 2.5 cm. Maximal voluntary contraction (MVC) was calculated by taking the average of the two highest (three total trials with 1 min between each) MVCs and was used to calculate 20% MVC. Subjects underwent a randomized‐crossover design and performed two exercise bouts at either 20% (low) or 50% (high) duty cycle, described previously (Broxterman et al. 2014). Briefly, the low duty cycle matches the high duty cycle's concentric contraction time (0.6 sec) (Abbott et al. 1952; Ryschon et al. 1997). However, the low duty cycle excludes the isometric transition phase (0.3 sec), and the eccentric relaxation phase (0.6 sec) [Fig. 1]. To best control for potential differences in metabolic rate across each duty cycle, work rate (20% MVC) and contraction frequency (20 contractions min^−1^) were kept constant. After preliminary data were analyzed a second experimental day was performed to determine the impact of relaxation time at a different metabolic rate, by performing the experiment at 15% MVC with the low duty cycle. The results of resting data for day two showed an identical vasoconstrictor response and were excluded from analysis to reduce redundancy of results.

Prior to the initiation of handgrip exercise, a 2‐min baseline coupled with 2‐min of LBNP (~ 30 mmHg) were completed to establish a “resting” vasoconstrictor response (i.e., no functional sympatholysis). Next, a 2‐min baseline and 7‐min handgrip protocol was performed. During the final 2 min of handgrip exercise LBNP was used to observe changes in the “exercising” vasoconstrictor response (i.e., functional sympatholysis). After the first bout of handgrip exercise, a minimum of 10‐min recovery was given to allow forearm blood flow (FBF) and blood pressure to return back to steady baseline values (Shoemaker et al. 1997). This was confirmed by similar, blood pressure and brachial artery velocity profiles and the handgrip protocol was performed again.

### Data analysis

All data were time aligned and averaged into 1‐min bins during resting and steady‐state exercise measurements. FBF was calculated as: FBF = mean blood velocity • 60 • *π* • (brachial diameter/2)^2^ calculated in mL min^−1^. Importantly, MAP was time aligned with FBF to calculate forearm vascular conductance (FVC) calculated in mL min^−1^ 100 mmHg [FVC = (FBF/MAP)*100]. At rest, FBF, FVC, and MAP measurements were averaged across the second minute (min 1–2) of rest and LBNP (min 3–4). During exercise, FBF, FVC, and MAP were calculated as a minute average prior to LBNP (steady‐state: min 4–5) and during the final minute of LBNP (min 6–7). Functional sympatholysis was calculated as: %∆FVC = FVC_LBNP_–FVC_ss_)/FVC_ss_ × 100, where “ss” denotes steady‐state (Buckwalter and Clifford 2001; Schrage et al. 2005). Following data collection, NIRS data were averaged into 1‐min epochs for deoxy‐[heme] and total‐[heme], which were time aligned with blood flow and blood pressure. In addition, Craig et al. 2017 used an adjustment to the NIRS output to adequately return signal values to original [Heme] concentrations and the NIRS signals were multiplied by 4. The original syntax used for the NIRS was based on brain oxygenation (key chromophore is Hb) and the signal was divided by 4 in the software. It is now known that [Mb] plays a significant role in the skeletal muscle oxygenation (Davis and Barstow 2013) and must have this correction applied. The new concentration agrees with previous data of muscle biopsy [Mb] when transformed into appropriate units (Reynafarje 1962).

### Estimation of forearm oxygen consumption

To quantify forearm metabolic rate, $\dot{V}O_{2}$ was calculated as a function of brachial artery blood flow and deoxy‐[heme], a proxy for arterial‐venous O_2_ difference (Delorey et al. 2003; Grassi et al. 2003), as described previously (Davis and Barstow 2013; Broxterman et al. 2014). The deoxy‐[heme] values are in *μ*mol heme/l tissue, and tissue is assumed to be metabolically active skeletal muscle. The deoxy‐[heme] values can be converted into *μ*mol heme/l blood using a conversion of 1.36% capillary blood volume/muscle volume (taken from 400 cap/mm^2^, 28.2 *μ*m^2^ cross‐sectional area, and a coefficient of 1.2 which corrects for the tortuosity and branching of the capillaries) (Richardson et al. 1994). These units can then be converted using specific units (mole O_2_/L blood) assuming that 1 mole O_2_/mole heme, and further to LO_2_/L blood using the conversion 22.4 L O_2_/mole O_2_. $\dot{V}O_{2}$ values in LO_2_/min may then be obtained by simply multiplying this value by the measured brachial artery blood flow.

### Statistics

Data were analyzed with commercially available statistical software package (Sigmaplot; version 12.5, Systat software, San Jose). MAP, FBF, FVC, deoxy‐[heme], total‐[heme], and$\dot{V}O_{2}$ were analyzed with a two‐way repeated measures ANOVA with a Bonferroni correction for pairwise comparison. The level of significance was set at (*P *< 0.05). All data were presented as means ± standard error.

## Results

### Steady‐state exercise hemodynamic responses

Significant interactions (time × condition) were present for FBF and FVC, and $\dot{V}O_{2}$ (*P *< 0.01), but not MAP (*P* =  0.07) or NIRS derived variables (all: *P* =  0.10). Pairwise tests revealed that FBF and FVC during the low duty cycle at 20% MVC were significantly greater than the high duty cycle (20% MVC) and low duty cycle (15% MVC) (Table 1; *P *< 0.01). The integration of FBF and deoxy‐[heme] yielded similar steady‐state $\dot{V}O_{2}$ response during the low and high duty cycles at 20% MVC (Table 2, P > 0.05). Importantly, the low duty cycle at 15% MVC had significantly lower $\dot{V}O_{2}$ compared to both duty cycles at 20% MVC (*P* = 0.02). Deoxy‐ and total‐[heme] NIRS variables were not significantly different between duty cycles (Table 2; *P* > 0.05).

**Table 1:** Hemodynamic response at rest and during exercise

	Forearm blood flow (mL min^−1^)	Mean arterial pressure (mmHg)	Forearm vascular conductance (mL min^−1^ Kg 100 mmHg)
Resting	84 ± 11^a^	88 ± 3	97 ± 13^a^
Resting + LBNP	51 ± 5^a^^,^ b	88 ± 2	59 ± 6^a^^,^ b
Low duty‐cycle (20% MVC)			
Steady‐state	392 ± 20^a^^,^ c	100 ± 4	398 ± 34^a^^,^ c
Steady‐state + LBNP	344 ± 26^a^^,^ b	100 ± 4	344 ± 26 a ^,^ c
High duty cycle (20% MVC)			
Steady‐state	330 ± 27^a^	103 ± 5	327 ± 37^a^
Steady‐state + LBNP	322 ± 18^a^	102 ± 4	320 ± 26^a^
Low duty‐ cycle (15% MVC)			
Steady‐state	282 ± 13^a^^,^ d	95 ± 3	301 ± 16^a^^,^ d
Steady‐state + LBNP	250 ± 10^a^^,^ b ^,^ d	96 ± 3	261 ± 12^a^^,^ b ^,^ d

Data are means ± standard error.

a Denotes significant main effect for time (*P* < 0.01).

b Denotes main effect of LBNP (*P* < 0.01).

c Denotes significant difference from the high duty cycle in same condition (*P* < 0.01).

d Significant difference from low duty cycle (20% MVC) in same condition (*P* < 0.01).

**Table 2:** Metabolic response: $\dot{V}O_{2}$_,_ deoxy‐[heme], and total‐[heme] derived from near‐infrared spectroscopy. ($\dot{V}O_{2}$) was the product of muscle blood flow and deoxy‐[heme] (2)

	*V*0_2_ (mL min^−^’)	Deoxy‐[heme] *μ*mol L^−1^	Total‐[heme] *μ*mol L^−1^
Resting	15.4 ± 5.3^c^	83.6 ± 5.2	338.0 ± 18.8
LBNP	338.0 ± 3.7^c^	98.8 ± 3.2	322.8 ± 18.0
Low duty cycle (20% MVC)			
Steady‐state	71.6 ± 6.7^c^	113.6 ± 8.0	387.6 ± 24.8
Steady‐state + LBNP	65.6 ± 72^c^	121.6 ± 9.6	382. ± 23.6
High duty cycle (20% MVC)			
Steady‐state	67.6 ± 5.8^c^	126.0 ± 10.8	367.2 ± 19.6
Steady‐state + LBNP	68.1 ± 4.1^c^	130.0 ± 10	363.6 ± 19.6
Low duty‐ cycle (15% MVC)			
Steady‐state	49.1 ± 7.9^c^^,^ d	100.4 ± 9.2	354.0 ± 20.8
Steady‐state + LBNP	43.4 ± 6.9^c^^,^ d	100.4 ± 9.6	352.4 ± 20.4

Data are means ± standard error.

Denotes significant main effect for time (*P *< 0.01).

Denotes significantly lower than 20% MVC conditions (*P *< 0.01).

### Steady‐state exercise + LBNP hemodynamic responses

Pairwise tests revealed a significant reduction in resting FBF and FVC with the application of LBNP (both; *P *< 0.01). During exercise, FBF and FVC were significantly decreased with LBNP during both low duty cycle conditions (*P *< 0.01: 20% MVC; *P* = 0.03: 15% MVC). However, there was no significant decrease in FVC or FBF during the high duty cycle (both: *P* > 0.05). Deoxy‐ and total‐[heme] NIRS variables were not significantly different between duty cycles during LBNP (Table 2; *P* > 0.05).

**Figure 2:**
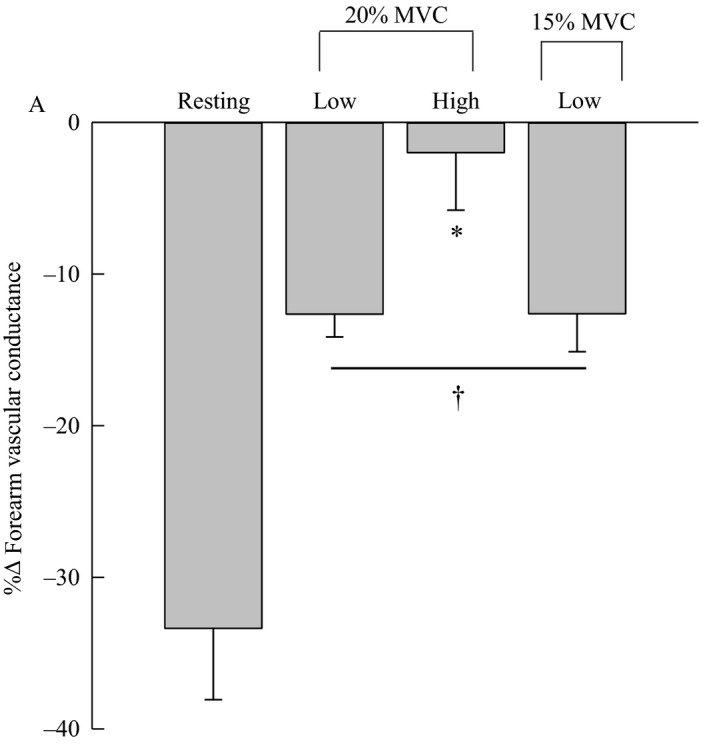
Percent change in forearm vascular conductance. * denotes significant difference from low duty cycles (*P *< 0.01). ^†^denotes significantly different from rest (*P *< 0.01). Data are means ± standard error. Low designates 20% duty cycle, high designates 50% duty cycle.

Figure 2 illustrates the calculated functional sympatholysis response for each condition. The %ΔFVC was significantly attenuated by exercise in all conditions (Fig. 2; *P *< 0.01). The %ΔFVC during both low duty cycle was significantly lower than the high duty cycle (Fig. 2; *P* = 0.01; *P* = 0.03, respectively). There was no significant difference between %ΔFVC during the low duty cycles (*P* = 0.99). As previously mentioned in the methods, data from day two were not analyzed due to identical responses to day one (day 1: %ΔFVC: −33.36%; day 2: %ΔFVC: −34.33%).

## Discussion

The major new finding of the current investigation is that decreasing mechanical impedance, allowing for an increased relaxation time, altered vasoconstriction responses in the active skeletal muscle. We show an ~10% greater vasoconstrictor response during the low duty cycles compared to the high duty cycle, showing that the high duty cycle better attenuated LBNP evoked vasoconstriction. Interestingly, at a matched duty cycle, but different metabolic rate (i.e., 15% and 20% MVC), we demonstrate that functional sympatholysis was not significantly different (Fig. 2). These findings may suggest that when mechanical compression is minimized, increases in ${\dot{Q}}_{m}$ may play a role in functional sympatholysis.

In the current investigation, we demonstrate that a low duty cycle shifts the balance between vasoconstriction and vasodilation of the active skeletal muscle to a more pronounced vasoconstriction during LBNP (Fig. 2). We have demonstrated, during 20% MVC, that there is an ~10% larger vasoconstriction when mechanical impedance is decreased. Interestingly, even with the greater vasoconstriction, the low duty cycle was shown to have a bulk blood flow that was ~22 mL min^−1^ above the high duty cycle; however, this finding did not reach statistical significance (Table 1). Furthermore, it may be that the higher blood flow prior to LBNP was above that needed to maintain metabolic demand and a significant vasoconstriction was negligible. In a previous investigation it was shown, with intravital microscopy, that reducing perfusion attenuated norepinephrine‐mediated vasoconstriction (McGillivray‐Anderson and Faber 1991). As such, our current findings suggest that increased ${\dot{Q}}_{m}$ increased LBNP evoked vasoconstriction. However, this study is limited in speculating on specific mechanism(s) influencing the vasoconstrictor response, but may be linked to increased metabolite clearance and/or improvements in oxidative metabolism (McGillivray‐Anderson and Faber 1990, 1991; Wray et al. 2009).

During 20% MVC, functional sympatholysis was greater during the high duty cycle compared to the low duty cycle (Fig. 2). This agrees with our hypothesis and raises another important point; when work rate (20% MVC), and contraction frequency (20/min) are matched, differences in mechanical impedance and time under tension likely influenced the results (Richards et al. 2012; Bentley et al. 2017). For example, Bentley et al. (2017), showed that greater mechanical impedance, like that shown with the high duty cycle, will briefly impair blood flow resulting in a “rebound” vasodilation to acutely increase ${\dot{Q}}_{m}$. As such, it is likely that mechanical impedance influenced this rebound effect during the relaxation phase, potentially influencing functional sympatholysis. In theory, the increased mechanical impedance may have augmented the vasoactive metabolites, and functional sympatholysis, within the active musculature (Richards et al. 2012). Yet, muscle metabolites were not measured within the present investigation and further interpretation is limited.

Contrary to our hypothesis, the low duty cycle at 15% MVC did not show an increase in LBNP evoked vasoconstriction when compared to the low duty cycle at 20% MVC condition. This is an interesting point given that the low duty cycle at 15% MVC had a significantly lower $\dot{V}O_{2}$ compared to the low duty cycle at 20% MVC. These data suggest that metabolic rate can differ and still show a similar LBNP evoked vasoconstriction responses. While the majority of evidence suggests that functional sympatholysis largely is driven by metabolic rate (Buckwalter and Clifford 2001; Tschakovsky et al. 2002; Schrage et al. 2005; Kruse et al. 2017), we provide the first evidence that duty cycle caused a similar degree of vasoconstriction during different metabolic rates. It remains unknown why our data go against this previous work, but may be due to the similar microvascular tissue oxygenation (i.e., deoxy‐[heme], total‐[heme]), as determined by NIRS, between the conditions, suggesting that in our experimental set‐up there was an adequate delivery of muscle blood flow for the given metabolic demand (Ferreira et al. 2006).

A strength of the current investigation was the maintenance of contraction frequency (20 contractions/min), duration (3 sec), and intensity (20% MVC) during dynamic handgrip exercise. This led to similar estimates of $\dot{V}O_{2}$ between both duty cycles at 20% MVC (Table 2), and a lower $\dot{V}O_{2}$ at 15% MVC. This is important given that Kruse et al. (2017) demonstrated that vasoconstrictor responses were independent of contractile “work” and dependent on metabolic rate by altering contraction frequency (20 contractions/min vs. 10 contractions/min). Our results extend that of Kruse et al. (2017) by demonstrating that duty cycle and the subsequent changes in ${\dot{Q}}_{m}$, may also directly alter functional sympatholysis. Importantly, we show that the low duty cycles had a similar level vasoconstriction at different metabolic rates. As mentioned above, it may be that a relative overperfusion allowed a certain degree of vasoconstriction to take place, but more work in this area is needed. Taken together, in populations that are blood flow limited, lowering the duty cycle may be a viable option to improve ${\dot{Q}}_{m}$ (Poole et al. 2012).

### Experimental considerations

The current investigation used LBNP to increase sympathetic outflow to the periphery. As such, it cannot be determined if pre‐or‐post junctional adrenergic receptors were mediating the responses found in this study. Second, ${\dot{Q}}_{m}$, and deoxy‐[heme] from the NIRS were used to calculate $\dot{V}O_{2}$ during handgrip exercise with the description of this discussed in the methods. As such, the accuracy of $\dot{V}O_{2}$ may be hindered by assumptions used but were held constant to limit influence of the assumptions on detected changes found (2). Two assumptions were made to calculate $\dot{V}O_{2}$ with NIRS: (1) it was assumed that the deoxy‐[heme] signal is a reflection of only hemoglobin; however, it is known myoglobin plays a significant role (Davis and Barstow 2013); and (2) it was also assumed that the entire NIRS signal came from muscle without any impact of adipose tissue in the forearm. Moreover, the values obtained for $\dot{V}O_{2}$ in this study are similar to the direct measurements shown previously (Nyberg et al. 2017). Finally, the metabolic rate ($\dot{V}O_{2}$) was influenced by the different time under tensions when comparing between each duty cycle. This would directly impact the adenosine triphosphate (ATP) cost of contraction and must be considered with regard to the findings presented.

## Conclusion

The current investigation has demonstrated that the ability to attenuate sympathetically mediated vasoconstriction (i.e., functional sympatholysis), during muscular contraction may shift toward an increased vasoconstriction if the contraction–relaxation cycle (i.e., duty cycle) is manipulated to lower mechanical impedance and increase ${\dot{Q}}_{m}$. Moreover, it was interesting to note that a low duty cycle at 15% MVC did not elicit a larger vasoconstriction relative to the 20% MVC condition, suggesting adequate delivery of muscle blood flow for the given metabolic demand. This investigation highlights the potential for utilizing a lower duty cycle to improve bulk ${\dot{Q}}_{m}$ in individuals with compromised transport. This may allow individuals to sustain a higher exercise intensity and/or increase exercise tolerance.

## Conflict of Interests

None declared.
